# Loneliness and Social Isolation Factors Under the Prolonged COVID-19 Pandemic in Japan: 2-Year Longitudinal Study

**DOI:** 10.2196/51653

**Published:** 2024-09-09

**Authors:** Nagisa Sugaya, Tetsuya Yamamoto, Naho Suzuki, Chigusa Uchiumi

**Affiliations:** 1 National Institute of Occupational Safety and Health, Japan Kawasaki Japan; 2 Graduate School of Technology, Industrial and Social Sciences, Tokushima University Tokushima Japan; 3 Department of Psychology, Keio University Tokyo Japan

**Keywords:** COVID-19, pandemic, loneliness, social isolation, longitudinal survey, epidemiology, mental health

## Abstract

**Background:**

Worsening loneliness and social isolation during the COVID-19 pandemic have become serious public health concerns worldwide. Despite previous research reporting persistent loneliness and social isolation under repeated emergency declarations and prolonged pandemics, long-term studies are needed to identify the actual conditions of loneliness and social isolation, and the factors that explain them.

**Objective:**

In this study, 3 web-based surveys were conducted at 1-year intervals during the 2 years after the first state of emergency to examine changes in loneliness and social isolation and the psychosocial factors associated with them in the Japanese population.

**Methods:**

The first survey (phase 1, May 11-12, 2020) was conducted at the end of the first emergency declaration period, the second survey (phase 2, June 14-20, 2021) was conducted at the end of the third emergency declaration period, and the third survey (phase 3, May 13-30, 2022) was conducted when the state of emergency had not been declared but many COVID-19–positive cases occurred during this period. We collected data on 3892 inhabitants (n=1813, 46.58% women; age: mean 50.3, SD 13.4 y) living in the 4 prefectures where emergency declaration measures were applied in phases 1 and 2. A linear mixed model analysis was performed to examine the association between psychosocial variables as explanatory variables and loneliness scores as the dependent variable in each phase.

**Results:**

While many psychosocial and physical variables showed improvement for the 2 years, loneliness, social isolation, and the relationship with familiar people deteriorated, and the opportunities for exercise, favorite activities, and web-based interaction with familiar people decreased. Approximately half of those experiencing social isolation in phase 1 remained isolated throughout the 2-year period, and a greater number of people developed social isolation than those who were able to resolve it. The results of the linear mixed model analysis showed that most psychosocial and physical variables were related to loneliness regardless of the phase. Regarding the variables that showed a significant interaction with the phase, increased altruistic preventive behavior and a negative outlook for the future were more strongly associated with severe loneliness in phase 3 (*P*=.01 to <.001), while the association between fewer social networks and stronger loneliness tended to be more pronounced in phase 2. Although the interaction was not significant, the association between reduced face-to-face interaction, poorer relationships with familiar people, and increased loneliness tended to be stronger in phase 3.

**Conclusions:**

This study found that loneliness and social isolation remained unresolved throughout the long-term COVID-19 pandemic. Additionally, in the final survey phase, these issues were influenced by a broader and more complex set of factors compared to earlier phases.

## Introduction

### Background

Since its outbreak in December 2019, COVID-19 has spread rapidly worldwide [1]. To deter its spread, many countries imposed repeated lockdowns, such as restricting people’s movement and temporarily closing services. However, while lockdowns were effective at preventing the spread of infection, they caused substantial financial hardship and psychological distress [2,3].

The lockdown and stay-at-home orders announced globally during the COVID-19 pandemic have led to physical and social distancing, and many individuals have experienced social isolation [1,4]. Increased loneliness during the stay-at-home period is strongly associated with severe depression and suicidal ideation [5,6]. However, the magnitude of social support during the pandemic was inversely associated with suicidal ideation and self-harm [7]. Thus, worsening loneliness and social isolation during the COVID-19 pandemic have become serious public health concerns.

In Japan, 4 states of emergency owing to the COVID-19 pandemic were declared between 2020 and 2021. While many countries were in lockdown with penalties for violations, a unique feature of Japan’s COVID-19 policy was that the government requested that people refrain from going out except in an emergency, certain businesses closed temporarily, and no penalties were imposed for violations. Since the declaration of a state of emergency in Japan was a *request* by the government, it did not prohibit going out or meeting people. However, as in other countries, Japan’s mild lockdown [8] affected people’s lives in many ways, including lifestyle changes due to teleworking and web-based classes, and economic hardships due to reduced income and unemployment. Our previous research reported severe loneliness, social isolation, and psychological distress during the state of emergency in Japan [4,9].

Several longitudinal studies have reported persistent problems of loneliness and social isolation during repeated emergency declarations and prolonged pandemics in Japan. In our longitudinal studies conducted during the pandemic (May 2020 and February 2021), whereby a state of emergency was declared in the Japanese population [10], there were no improvements in severe social isolation and loneliness between the 2 phases, although psychological distress significantly improved, and depression slightly decreased. Another longitudinal survey conducted during the latter half of the second wave and at the end of the fifth wave of the pandemic in Japan reported that from the first to the second year of the COVID-19 pandemic, social isolation (the evaluation method was different from the one we used) decreased but loneliness increased [11]. This study was not conducted under emergency conditions. The loneliness of the Japanese people was severe not only during the period when the state of emergency was declared but also during other periods when the COVID-19 infection spread.

### Objectives

**Figure 1:**
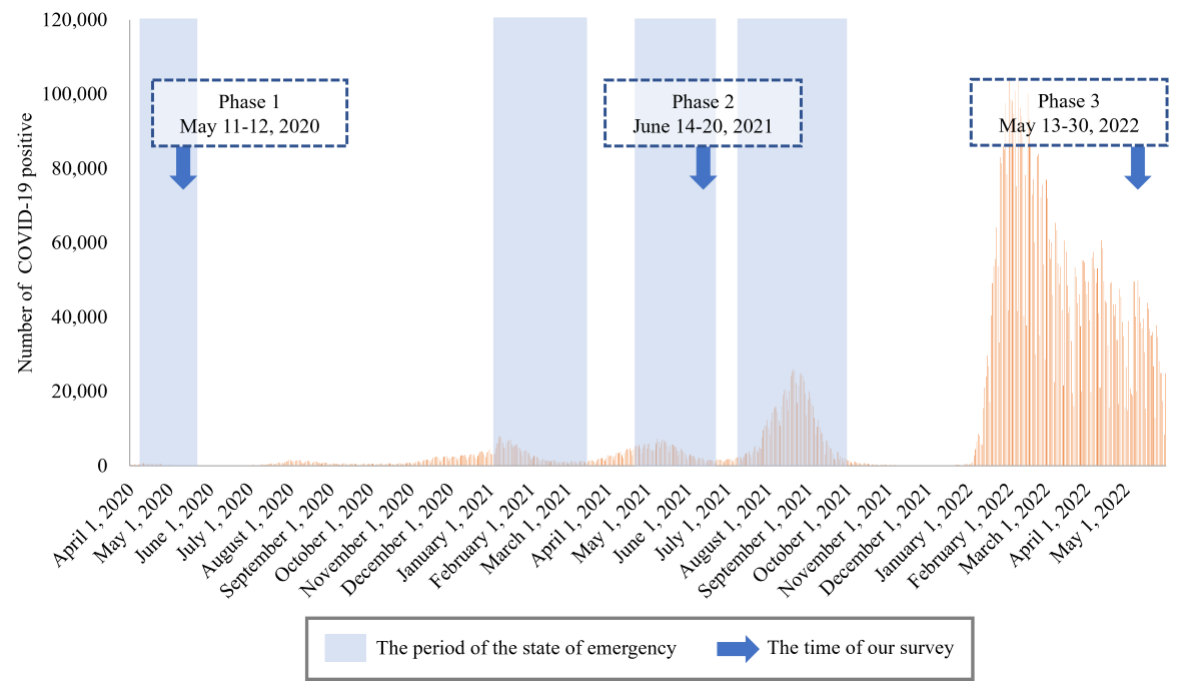
Change in the number of COVID-19–positive cases in Japan between phases 1 and 3.

Unlike other psychological variables, loneliness and social isolation during a pandemic are unlikely to improve through longitudinal observation. Therefore, a long-term study is needed to identify the actual conditions of loneliness and social isolation and the factors that explain them. In this study, 3 surveys were conducted at 1-year intervals during the 2 years after the first state of emergency to examine changes in loneliness and social isolation and the psychosocial factors associated with them. The first survey (phase 1, May 11-12, 2020) was conducted at the end of the first emergency declaration period, the second survey (phase 2, June 14-20, 2021) was conducted at the end of the third emergency declaration period, and the third survey (phase 3, May 13-30, 2022) was conducted when the state of emergency was not declared but many COVID-19–positive cases occurred during this period (Figure 1). This study will enable us to observe changes in loneliness and social isolation across long-term periods as well as changes in these variables with changes in social conditions, such as the declaration of a state of emergency, thereby providing useful information for considering when and which factors to intervene in during a prolonged pandemic.

## Methods

### Participants and Data Collection

Web-based surveys were conducted between May 11 and 12, 2020 (phase 1); June 14 and 20, 2021 (phase 2); and May 13 and 30, 2022 (phase 3). The first and third states of emergency were declared in phases 1 and 2, respectively. In phase 1, we conducted a web-based survey of inhabitants living in 7 prefectures where emergency measures were first applied (Tokyo, Kanagawa, Osaka, Saitama, Chiba, Hyogo, and Fukuoka) to precisely detect the impact of the declaration. We recruited participants according to the following inclusion criteria: (1) inhabitants living in the 7 prefectures mentioned above and (2) aged ≥18 years. The exclusion criteria were as follows: (1) aged <18 years, (2) high-school students, and (3) living outside the 7 prefectures. In phase 1, 11,333 individuals participated and the number of people in each prefecture was determined based on the ratio of the number of people living in Tokyo (n=2783, 24.56%), Kanagawa (n=1863, 16.44%), Osaka (n=1794, 15.83%), Saitama (n=1484, 13.09%), Chiba (n=1263, 11.14%), Hyogo (n=1119, 9.87%), and Fukuoka (n=1027, 9.06%). We conducted a follow-up survey on the same participants in phase 2 but excluded inhabitants living in Kanagawa, Saitama, and Chiba, where the emergency declaration was not applied from the survey in phase 2. In phase 2, we collected data from 4592 individuals who lived in Tokyo, Osaka, Hyogo, and Fukuoka and had participated in survey phase 1. In phase 3, 3892 participants who participated in phases 1 and 2 responded to an additional follow-up survey.

Study participants were recruited through Macromill Inc (Tokyo, Japan), a global marketing research company. This company has access to >1,300,000 registered members, with diverse characteristics regarding sex and age, from all prefectures in Japan. The web-based survey system automatically eliminated duplicate answers from a single respondent. Approximately 80,000 registered people living in the target areas were recruited via email, and data were collected on a web-based platform. Participants completed the web-based survey after receiving a link. All participants voluntarily and anonymously responded to the survey and provided informed consent on the web before completing the survey. The participants received a clear explanation of the survey procedure and could discontinue or terminate the survey at any time without providing a reason. The questionnaire format, excluding the default items provided by Macromill Inc (sex, age, occupation, annual household income, marital status, and presence of children), did not allow participants to proceed to the next page if there were items they had not answered. All participants received Macromill points for their participation, which constituted an original point service of Macromill, Inc, and participants could exchange these points for prizes or cash.

The data in this phase of the study were partly extracted from a database containing the data used in our previous studies [12,13]. The extracted data were secondarily reanalyzed with dependent and independent variables different from those in the studies mentioned above. A detailed explanation of the data set used in this study is provided in the study by Sugaya et al [14].

### Measures

#### Sociodemographic Data

Sociodemographic information was collected on participants, including age, sex, employment status (employed, homemaker, student, unemployed, or other), marital status, and annual household income (<2.0 million JPY; 1 JPY=US $0.0093 as of May 11, 2020; 2.0-3.9 million JPY, 4.0-5.9 million JPY, 6.0-7.9 million JPY, ≥8.0 million JPY, or unknown). In addition, phases 2 and 3 included the number of cohabitants.

#### Loneliness

Loneliness was measured using the Japanese version of the University of California, Los Angeles Loneliness Scale Version 3 (UCLA-LS3) [15]. The UCLA-LS3 consists of 10 items rated from 1 (never) to 4 (always) [16]. Total scores ranged from 10 to 40, with higher scores indicating higher levels of loneliness.

#### Social Isolation

We measured social networks using the Japanese version of the abbreviated Lubben Social Network Scale-6 (LSNS-6) [17]. The LSNS-6 is a shortened version of the Lubben Social Network Scale [18], which includes items on the network size of relatives or friends who provide emotional and instrumental support. The LSNS-6 consists of 3 items related to family networks and 3 items related to friendship networks. The number of people in the network was calculated using a 6-point scale (0=none, 1=one, 2=two, 3=three or four, 4=five-eight, and 5=nine or more) for each item [19]. The total score ranges from 0 to 30 points, with higher scores indicating a larger social network and scores of <12 points indicating social isolation.

Loneliness and social isolation are conceptually distinct, with social isolation generally defined in terms of the objective availability of social contacts and the frequency of contact with social network members. Loneliness refers to the perception that personal and social needs are not being met [20,21]. Moreover, social isolation is reportedly related to loneliness and is often a risk factor [22].

#### Psychological Distress

We used the Japanese version of the Kessler Psychological Distress Scale-6 (K6) [23], a nonspecific psychological stress scale. The K6 is a 6-item screening instrument that measures distress over the past 30 days. Each question is rated on a scale of 0 (never) to 4 (always), with total scores ranging from 0 to 24. The K6 is considered an ideal instrument for screening mental disorders in population-based health surveys because of its brevity and high accuracy [23-25].

#### Depressive Symptoms

We used the Japanese version of the Patient Health Questionnaire-9 (PHQ-9) [26] to collect basic information on participants’ mental health; the PHQ-9 consists of 9 questions. Participants reported depressive symptoms during the past 4 weeks with a score of 0 (none) to 3 (nearly every day) [27].

#### Physical Symptoms

The Japanese version of the Somatic Symptom Scale-8 (SSS-8) was used to assess physical symptom burden [28]. The SSS-8 consists of 8 items that assess the following somatic symptoms: stomach or bowel problems; back pain; pain in the arms, legs, or joints; headaches; chest pain or shortness of breath; dizziness; feeling tired or having low energy; and trouble sleeping. These items comprise 4 symptom domains: gastrointestinal, pain, cardiopulmonary, and fatigue. Participants reported how much each symptom had bothered them during the previous 7 days, with a score of 0 to 4 (0=not at all, 1=a little bit, 2=somewhat, 3=quite a bit, and 4=very much) [29].

#### Lifestyle, Coping Behavior, and Stressors Related to COVID-19 Pandemic

With extensive references to the literature on the COVID-19 pandemic [30-34], we developed 8 lifestyle and coping behavior items, and 7 stressors were assumed to be associated with the COVID-19 pandemic (Multimedia Appendix 1) [8,12]. We asked participants to rate the frequency of implementation and their experience of these items from the start of the state of emergency (phases 1 and 2) or the last 30 days (phase 3) to the time of the survey on a scale of 1 (not at all) to 7 (extremely). The item details are described in our previously published articles [8,12].

### Statistical Analysis

ANOVA was applied to compare the psychosomatic variables and items of the COVID-19 pandemic between the phases. The chi-square test was used to compare sociodemographic data between the social isolation groups (participants were assigned to each group based on their LSNS-6 scores). Repeated 2-way ANOVA was conducted to confirm the interactions between demographic characteristics and phases on the UCLA-LS3 score. We applied a linear mixed model to effectively analyze the diverse variables and data from the 3 time points. To select variables for the linear mixed model, multiple regression analysis using the stepwise method was performed with the UCLA-LS3 score as the dependent variable and the other variables as explanatory variables in each phase. Variables significantly associated with the UCLA-LS3 score in the multiple regression analysis were used as explanatory variables in the linear mixed model analysis, which was conducted to examine the association between these explanatory variables and the UCLA-LS3 score as the dependent variable in each phase, with the participants as random effect. For all tests, significance was set at α=.05, using a 2-tailed approach. Statistical analyses were performed using SPSS Statistics (version 29.0; IBM Corp).

### Ethical Considerations

This study was approved by the research ethics committee of the Graduate School of Social and Industrial Science and Technology at Tokushima University (approval no. 212). The survey procedures were clearly explained, and participants could interrupt or terminate the survey at any time without providing an explanation. This study was conducted in accordance with the ethical standards of the 1964 Declaration of Helsinki and its subsequent amendments.

## Results

### Descriptive Results

Table 1 shows the sociodemographic characteristics of the participants. A total of 3892 individuals participated in phases 1, 2, and 3 (n=1813, 46.58% women; mean age 50.3, SD 13.4; range 18-89 years in phase 1). However, 42.11% (2831/6723) of the individuals who lived in Tokyo, Osaka, Hyogo, and Fukuoka and who participated in phase 1 did not respond in phases 2 or 3. In addition, there were significantly more females than males among individuals who did not participate in phases 2 or 3. Individuals who did not participate in phase 2 or 3 were younger and had lower UCLA-LS3 scores and higher LSNS-6, K6, PHQ-9, and SSS-8 scores than those who participated in the 3 phases.

**Table 1:** Change in psychosocial and physical variables during 3 phases.

Variables	Phase 1, mean (SD)	Phase 2, mean (SD)	Phase 3, mean (SD)	*F* (*df*)	*P* value	η^2^
UCLA-LS3^a^	23.6 (5.8)	24.0 (5.9)	23.9 (6.0)	21.48 (1.98, 7708.59)	<.001	0.005
LSNS-6^b^	10.0 (6.1)	9.4 (6.0)	9.3 (6.0)	42.94 (1.98, 7711.90)	<.001	0.011
K6^c^	5.1 (5.3)	4.0 (5.2)	3.9 (5.4)	167.66 (1.97, 7678.34)	<.001	0.041
PHQ-9^d^	4.4 (5.4)	3.9 (5.4)	3.8 (5.4)	44.05 (1.97, 7679.82)	<.001	0.011
SSS-8^e^	6.3 (5.5)	5.1 (5.5)	5.1 (5.5)	187.41 (1.94, 7538.18)	<.001	0.046
Exercise	4.1 (1.8)	3.8 (1.9)	3.8 (1.9)	95.75 (1.98, 7716.06)	<.001	0.024
Healthy eating habits	4.3 (1.5)	4.2 (1.6)	4.2 (1.6)	19.89 (2.00, 7782.00)	<.001	0.005
Healthy sleep habits	4.7 (1.7)	4.7 (1.7)	4.8 (1.7)	2.21 (1.99, 7726.92)	.11	0.001
Favorite activity	4.0 (1.6)	3.7 (1.7)	3.8 (1.7)	53.23 (2.00, 7766.41)	<.001	0.013
Offline interaction with familiar people	3.5 (1.8)	3.3 (1.8)	3.9 (1.8)	177.67 (1.99, 7751.62)	<.001	0.044
Web-based interaction with familiar people	3.1 (1.9)	2.7 (1.7)	2.8 (1.8)	90.16 (1.98, 7700.56)	<.001	0.023
Altruistic preventive behavior	5.4 (1.7)	5.4 (1.7)	5.4 (1.7)	3.89 (1.99, 7728.86)	.02	0.001
Optimism	4.0 (1.5)	4.2 (1.5)	4.3 (1.5)	69.17 (1.99, 7747.56)	<.001	0.017
Deterioration of household economy	3.7 (1.8)	3.5 (1.7)	3.5 (1.7)	35.68 (1.97, 7659.63)	<.001	0.009
Deterioration of relationship with familiar people	2.4 (1.5)	2.6 (1.6)	2.6 (1.5)	61.53 (2.00, 7782.00)	<.001	0.016
Frustration	3.2 (1.7)	3.2 (1.7)	3.1 (1.7)	4.43 (1.99, 7737.40)	.01	0.001
COVID-19–related anxiety	3.9 (1.7)	3.4 (1.7)	3.2 (1.6)	324.70 (1.98, 7714.55)	<.001	0.077
COVID-19–related sleeplessness	2.5 (1.5)	2.5 (1.5)	2.4 (1.5)	5.58 (1.99, 7737.59)	.004	0.001
Difficulties owing to the lack of daily necessities	3.5 (1.8)	2.5 (1.5)	2.5 (1.5)	684.09 (1.85, 7187.38)	<.001	0.150
Difficulties in work or schoolwork	3.6 (2.0)	2.8 (1.7)	2.7 (1.7)	465.29 (1.91, 7428.28)	<.001	0.107

^a^UCLA-LS3: University of California, Los Angeles Loneliness Scale Version 3.

^b^LSNS-6: Lubben Social Network Scale-6.

^c^K6: Kessler Psychological Distress Scale-6.

^d^PHQ-9: Patient Health Questionnaire-9.

^e^SSS-8: Somatic Symptom Scale-8.

In the comparisons of psychosocial and physical variables between phases, there were significant differences between phases in all variables except “healthy sleep habits.” Regarding the UCLA-LS3, “healthy eating habits,” “altruistic preventive behavior,” “deterioration of household economy,” “frustration,” and “COVID-19-related sleeplessness” results did not exceed the lower limit of “small effect size” (η^2^≥0.010). While many indicators showed improvement from phase 1 to phase 3, there were increases in the UCLA-LS3 scores and “deterioration of relationship with familiar people” and decreases in the LSNS-6 scores, “exercise,” “favorite activity,” and “web-based interaction with familiar people.”

### Transition of the Presence of Social Isolation During 3 Phases

**Figure 2:**
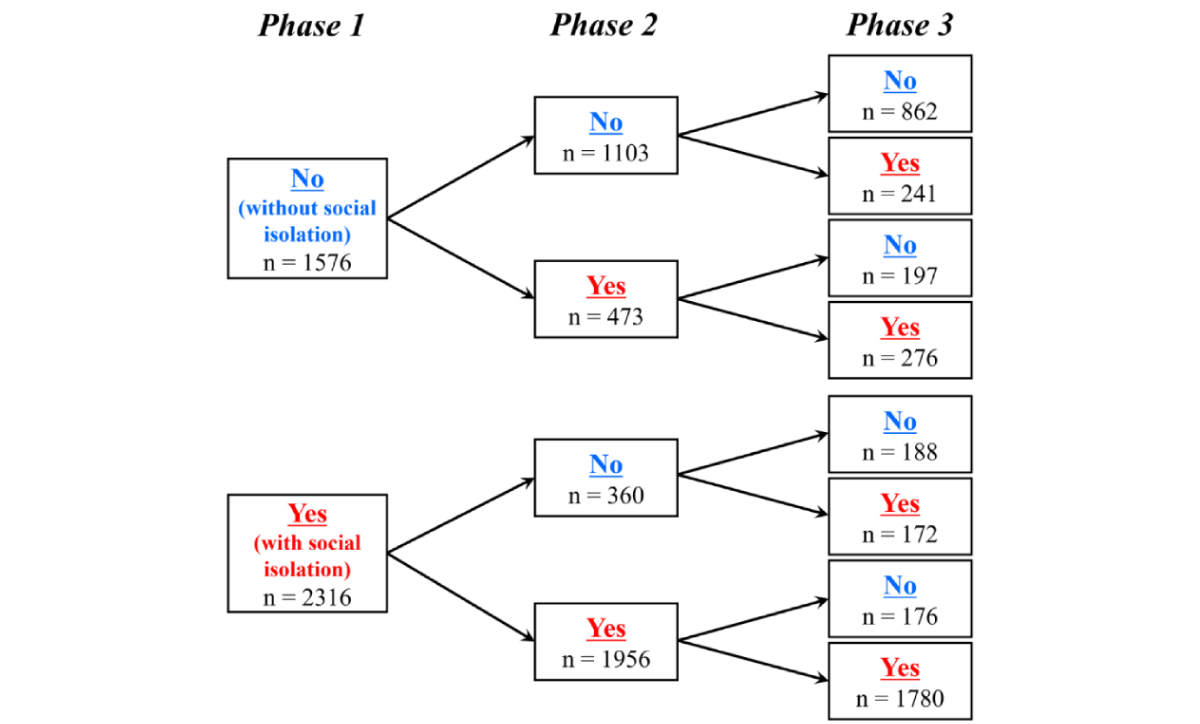
Transition of the presence of social isolation during the 3 phases (N=3892); no: Lubben Social Network Scale (LSNS-6) ≥12 (individuals without social isolation); yes: LSNS-6 <12 (individuals with social isolation).

Figure 2 shows the transitions in the presence of social isolation during the 3 phases. Of the 2316 individuals who were socially isolated in phase 1, 360 (15.54%) were no longer socially isolated in phase 2, whereas 473 (30.01%) of the 1576 individuals who were not socially isolated in phase 1 became socially isolated in phase 2. Of the 2429 persons who were socially isolated in phase 2, 373 (15.36%) were no longer socially isolated in phase 3, whereas 413 (28.23%) of the 1463 persons who were not socially isolated in phase 2 became socially isolated in phase 3. In addition, 45.73% (1780/3892) of the total respondents were consistently socially isolated in all 3 phases.

### Social Isolation and Psychosocial and Physical Variables in 3 Phases

Table 2 shows the number of individuals experiencing social isolation based on sociodemographic characteristics between the 3 phases. Regarding the sex differences, more males than females were socially isolated in all phases (phase 1: *χ*^2^_1_=24.7; *P*<.001; phase 2: *χ*^2^_1_=18.3; *P*<.001; phase 3: *χ*^2^_1_=21.9; *P*<.001). In all phases, fewer people were socially isolated in the ≥65 years group and more people were socially isolated in the 50 to 64 age group; in phase 1 and phase 3, fewer people were socially isolated in the 18 to 29 age group (phase 1: *χ*^2^_3_=33.9; *P*<.001; phase 2: *χ*^2^_3_=32.6; *P*<.001; phase 3: *χ*^2^_3_=46.2; *P*<.001). Regarding occupation, in all phases, fewer homemakers were socially isolated and greater number of people were unemployed; in phases 1 and 2, there were more socially isolated individuals in the other occupation group than in either occupation groups (*χ*^2^_4_=28.2; *P*<.001; phase 2: *χ*^2^_4_=37.1; *P*<.001; phase 3: *χ*^2^_4_=38.8; *P*<.001). A greater number of unmarried individuals were socially isolated than married individuals in all 3 phases (phase 1: *χ*^2^_1_=100.1; *P*<.001; phase 2: *χ*^2^_1_=117.9; *P*<.001; phase 3: *χ*^2^_1_=97.7; *P*<.001). In every phase, individuals without children were more likely to be socially isolated (*χ*^2^_1_=119.6; *P*<.001; phase 2: *χ*^2^_1_=161.9; *P*<.001; phase 3: *χ*^2^_1_=147.9; *P*<.001). Regarding annual household, in all phases, there were more socially isolated people in the <JPY 2.0 million group and JPY 2.0-3.9 million group, and fewer in the >JPY 8.0 million group (phase 1: *χ*^2^_4_=111.7; *P*<.001; phase 2: *χ*^2^_4_=92.9; *P*<.001; phase 3: *χ*^2^_4_=106.7; *P*<.001).

**Table 2:** Social isolation and sociodemographic characteristics in each phase.

Sociodemographic indexes			Phase 1, n (%)						Phase 2, n (%)							Phase 3, n (%)				
			Total		With social isolation		Without social isolation		Total		With social isolation		Without social isolation		Total			With social isolation		Without social isolation
Overall			3892 (100)		2316 (59.51)		1576 (40.49)		3892 (100)		2429 (62.41)		1463 (37.59)		3892 (100)			2469 (63.44)		1423 (36.56)
**Sex**																				
	Male	2079 (100)		1313 (63.15)		766 (36.84)		2079 (100)		1362 (65.51)		717 (34.49)		2079 (100)			1387 (66.71)		692 (33.29)	
	Female	1813 (100)		1003 (55.32)		810 (44.7)		1813 (100)		1067 (58.85)		746 (41.15)		1813 (100)			1082 (59.68)		731 (40.32)	
**Age (y)**																				
	18-29	273 (100)		134 (49.08)		139 (50.92)		224 (100)		132 (58.93)		92 (41.07)		186 (100)			102 (54.84)		84 (45.16)	
	30-49	1575 (100)		954 (60.57)		621 (39.43)		1514 (100)		966 (63.80)		548 (36.20)		1448 (100)			921 (63.60)		527 (36.39)	
	50-64	1395 (100)		885 (63.44)		510 (36.56)		1454 (100)		956 (65.75)		498 (34.25)		1510 (100)			1035 (68.54)		475 (31.46)	
	≥65	649 (100)		343 (52.85)		306 (47.15)		700 (100)		375 (53.57)		325 (46.43)		748 (100)			411 (54.95)		337 (45.05)	
**Occupation**																				
	Employed	2693 (100)		1607 (59.67)		1086 (40.33)		2677 (100)		1682 (62.83)		995 (37.17)		2693 (100)			1728 (64.17)		965 (35.83)	
	Homemaker	585 (100)		308 (52.65)		277 (47.35)		594 (100)		321 (54.04)		273 (45.96)		566 (100)			301 (53.18)		265 (46.82)	
	Student	46 (100)		21 (45.65)		25 (54.35)		27 (100)		12 (44.44)		15 (55.56)		19 (100)			10 (52.63)		9 (47.37)	
	Unemployed	443 (100)		294 (66.37)		149 (33.63)		480 (100)		328 (68.33)		152 (31.67)		564 (100)			395 (70.04)		169 (29.96)	
	Other	125 (100)		86 (68.80)		39 (31.20)		114 (100)		86 (75.44)		28 (24.56)		50 (100)			35 (70)		15 (30)	
**Marital status**																				
	Married	2489 (100)		1334 (53.60)		1155 (46.40)		2500 (100)		1403 (56.12)		1097 (43.88)		2490 (100)			1437 (57.71)		1053 (42.29)	
	Unmarried	1403 (100)		982 (69.99)		421 (30.01)		1392 (100)		1026 (73.71)		366 (26.29)		1402 (100)			1032 (73.61)		370 (26.39)	
**The presence of child**																				
	Yes	2175 (100)		1128 (51.86)		1047 (48.14)		2226 (100)		1199 (53.86)		1027 (46.14)		2224 (100)			1230 (55.3)		994 (44.7)	
	No	1717 (100)		1188 (69.19)		529 (30.81)		1666 (100)		1230 (73.83)		436 (26.17)		1668 (100)			1239 (74.28)		429 (25.72)	
**The number of cohabitants**																				
	0	—^a^		—		—		467 (100)		353 (75.59)		114 (24.41)		635 (100)			488 (76.85)		147 (23.15)	
	1	—		—		—		1212 (100)		829 (68.40)		383 (31.60)		1156 (100)			775 (67.04)		381 (32.96)	
	2	—		—		—		1003 (100)		628 (62.61)		375 (37.39)		949 (100)			609 (64.17)		340 (35.83)	
	3	—		—		—		753 (100)		405 (53.78)		348 (46.22)		742 (100)			409 (55.12)		333 (44.88)	
	4	—		—		—		332 (100)		160 (48.19)		172 (51.81)		303 (100)			143 (47.19)		160 (52.81)	
	≥5	—		—		—		125 (100)		54 (43.20)		71 (56.80)		107 (100)			45 (42.06)		62 (57.94)	
**Annual household income (in million; JPY^b^)**																				
	<2.0	223 (100)		183 (82.06)		40 (17.94)		247 (100)		195 (78.95)		52 (21.05)		333 (100)			262 (78.68)		71 (21.32)	
	2.0-3.9	737 (100)		498 (67.57)		239 (32.43)		765 (100)		544 (71.11)		221 (28.89)		756 (100)			539 (71.30)		217 (28.70)	
	4.0-5.9	775 (100)		461 (59.48)		314 (40.52)		802 (100)		479 (59.73)		323 (40.27)		754 (100)			478 (63.40)		276 (36.60)	
	6.0-7.9	525 (100)		305 (58.10)		220 (41.90)		515 (100)		317 (61.55)		198 (38.45)		517 (100)			309 (59.77)		208 (40.23)	
	≥8.0	819 (100)		394 (48.11)		425 (51.89)		821 (100)		428 (52.13)		393 (47.87)		846 (100)			437 (51.65)		409 (48.35)	

^a^Not available.

^b^1 JPY=US $0.0093.

Table 3 compares the psychosocial and physical variables between participants with and without social isolation in each phase. In phase 1, there were significant differences between participants with and without social isolation in psychosocial and physical variables, except for COVID-19–related anxiety, sleeplessness, and difficulties in work or schoolwork. In phase 2, there were significant differences between participants with and without social isolation in psychosocial and physical variables, except for COVID-19–related anxiety and sleeplessness, difficulties due to the lack of daily necessities, and difficulties in work or schoolwork. In phase 3, there were significant differences between participants with and without social isolation in all psychosocial and physical variables.

**Table 3:** Comparison of psychosocial and physical variables between participants with and without social isolation in each phase.

Variables	Phase 1						Phase 2						Phase 3				
	With social isolation, n (%)	Without social isolation, n (%)	*t* (*df*)	*P* value	Cohen *d*	With social isolation, n (%)		Without social isolation, n (%)	*t* (*df*)	*P* value	Cohen *d*	With social isolation, n (%)		Without social isolation, n (%)	*t* (*df*)	*P* value	Cohen *d*
UCLA-LS3^a^	25.83 (5.25)	20.29 (4.82)	33.43 (3890)	<.001	1.092	26.13 (5.43)		20.42 (4.89)	32.97 (3890)	<.001	1.091	25.98 (5.46)		20.33 (4.98)	32.07 (3890)	<.001	1.068
K6^b^	5.79 (5.64)	4.16 (4.52)	10.02 (3789.08)	<.001	0.314	4.56 (5.60)		2.92 (4.32)	10.28 (3661.34)	<.001	0.319	4.66 (5.79)		2.58 (4.21)	12.89 (3686.79)	<.001	0.395
PHQ-9^c^	5.22 (5.83)	3.30 (4.36)	11.74 (3855.48)	<.001	0.363	4.55 (5.93)		2.84 (4.34)	10.36 (3745.89)	<.001	0.318	4.57 (5.88)		2.43 (4.14)	13.23 (3737.54)	<.001	0.402
SSS-8^d^	6.74 (5.82)	5.63 (4.93)	6.43 (3707.46)	<.001	0.203	5.59 (5.79)		4.36 (4.95)	7.05 (3453.40)	<.001	0.225	5.66 (5.82)		4.05 (4.65)	9.47 (3504.85)	<.001	0.297
Exercise	3.81 (1.83)	4.61 (1.64)	14.32 (3603.65)	<.001	0.458	3.47 (1.88)		4.21 (1.81)	12.07 (3172.29)	<.001	0.396	3.58 (1.87)		4.30 (1.83)	11.73 (3017.41)	<.001	0.388
Healthy eating habits	4.03 (1.58)	4.76 (1.37)	15.39 (3667.01)	<.001	0.489	3.93 (1.62)		4.61 (1.46)	13.53 (3332.90)	<.001	0.436	3.97 (1.65)		4.66 (1.49)	13.36 (3213.80)	<.001	0.433
Healthy sleep habits	4.50 (1.8)	5.03 (1.61)	9.54 (3622.47)	<.001	0.305	4.50 (1.75)		5.1 (1.55)	11.05 (3370.19)	<.001	0.355	4.57 (1.69)		5.12 (1.53)	10.48 (3214)	<.001	0.339
Favorite activity	3.68 (1.67)	4.39 (1.50)	13.83 (3612.49)	<.001	0.442	3.45 (1.70)		4.08 (1.59)	11.76 (3238.94)	<.001	0.383	3.61 (1.70)		4.24 (1.61)	11.51 (3105.44)	<.001	0.377
Offline interaction with familiar people	3.16 (1.76)	4.02 (1.78)	14.83 (3358.57)	<.001	0.485	3.01 (1.74)		3.81 (1.73)	13.81 (3083.15)	<.001	0.457	3.53 (1.76)		4.63 (1.57)	20.19 (3251.28)	<.001	0.651
Web-based interaction with familiar people	2.63 (1.75)	3.75 (1.95)	18.38 (3141.14)	<.001	0.612	2.39 (1.62)		3.09 (1.84)	12.01 (2783.71)	<.001	0.410	2.52 (1.68)		3.28 (1.89)	12.48 (2695.64)	<.001	0.429
Altruistic preventive behavior	5.18 (1.85)	5.79 (1.41)	11.55 (3839.85)	<.001	0.359	5.22 (1.79)		5.74 (1.47)	9.84 (3534.29)	<.001	0.310	5.21 (1.76)		5.59 (1.53)	7.03 (3306.28)	<.001	0.225
Optimism	3.59 (1.55)	4.54 (1.35)	20.21 (3664.40)	<.001	0.643	3.85 (1.54)		4.68 (1.40)	16.82 (3890)	<.001	0.557	3.97 (1.51)		4.80 (1.41)	17.03 (3890)	<.001	0.567
Deterioration of household economy	3.74 (1.76)	3.61 (1.75)	2.41 (3890)	.02	0.079	3.50 (1.72)		3.36 (1.69)	2.59 (3890)	.010	0.086	3.66 (1.67)		3.30 (1.63)	6.69 (3025.16)	<.001	0.221
Deterioration of relationship with familiar people	2.52 (1.54)	2.16 (1.42)	7.53 (3551.79)	<.001	0.242	2.68 (1.56)		2.53 (1.57)	2.91 (3890)	.004	0.096	2.79 (1.56)		2.39 (1.48)	7.96 (3092.18)	<.001	0.261
Frustration	3.27 (1.73)	3.02 (1.67)	4.43 (3890)	<.001	0.145	3.21 (1.73)		3.07 (1.71)	2.46 (3890)	.014	0.081	3.25 (1.66)		2.82 (1.61)	8.05 (3890)	<.001	0.268
COVID-19–related anxiety	3.93 (1.7)	3.96 (1.67)	0.53 (3890)	.59	0.017	3.44 (1.69)		3.43 (1.64)	0.23 (3890)	.82	0.008	3.28 (1.61)		3.17 (1.61)	2.04 (3890)	.04	0.068
COVID-19–related sleeplessness	2.50 (1.53)	2.40 (1.52)	1.95 (3890)	.05	0.064	2.48 (1.52)		2.42 (1.50)	1.12 (3890)	.26	0.037	2.44 (1.46)		2.30 (1.45)	2.91 (2988.40)	.004	0.097
Difficulties owing to the lack of daily necessities	3.53 (1.81)	3.40 (1.78)	2.24 (3890)	.03	0.073	2.53 (1.54)		2.52 (1.55)	0.38 (3890)	.71	0.013	2.52 (1.51)		2.35 (1.49)	3.54 (3003.67)	<.001	0.117
Difficulties in work or schoolwork	3.53 (1.93)	3.60 (1.98)	1.17 (3890)	0.24	0.038	2.78 (1.68)		2.78 (1.70)	0.07 (3890)	.94	0.002	2.75 (1.66)		2.50 (1.63)	4.55 (3018.45)	<.001	0.151

^a^UCLA-LS3: University of California, Los Angeles Loneliness Scale Version 3.

^b^K6: Kessler Psychological Distress Scale-6.

^c^PHQ-9: Patient Health Questionnaire-9.

^d^SSS-8: Somatic Symptom Scale-8.

### Comparison of Loneliness Between Phases and Between Sociodemographic Characteristics

Table 4 shows interactions between phases and sociodemographic characteristics, and the main effects of each independent variable in the UCLA-LS3 score. There was a significant interaction between phases and sex in the UCLA-LS3 score. In all phases, the UCLA-LS3 scores in male participants were significantly higher than the scores in females (phase 1 and 3: *P*<.001; phase 2: *P*=.02); for both males and females (*P*<.001 in all phases), the UCLA-LS3 scores in phase 2 (male participants: *P*=.047; female participants: *P*<.001) and phase 3 (male participants: *P*=.03; female participants: *P*<.001) were significantly higher than the scores in phase 1. The main effects of group and time were significant for all demographic characteristics (main effect of phase for occupation: *P*=.002; other analyses: *P*<.001). Regarding age groups, the group of individuals aged >65 years had lower UCLA scores than all other age groups (*P*<.001), and the group of individuals aged 50 to 64 years had lower scores than the group of individuals aged 30 to 49 years old (*P*<.001). For occupation, the employed group had lower UCLA-LS3 scores than the other group that did not fit into any of the 4 categories (*P*=.03), and the homemaker group had lower scores than other occupation groups except students(*P*<.001). The unmarried group had higher UCLA-LS3 scores than the married group. Participants without children showed higher scores than those with children. Regarding annual household income, group <JPY 2.0 million had higher UCLA-LS3 scores than other household income groups (*P*<.001) and groups with >JPY 8.0 million showed lower scores than JPY 2.0 to 3.9 million (*P*<.001), 4.0 to 5.9 million (*P*=.002), and 6.0 to 7.9 million groups (*P*=.04).

**Table 4:** Comparison of loneliness between phases and between sociodemographic characteristics.

Sociodemographic indexes			Phase 1		Phase 2		Phase 3		Interaction										Group										Phase						
			Values, mean (SD)		Values, mean (SD)		Values, mean (SD)		*F* (*df*)			*P* value			η_p_^2^			*F* (*df*)			*P* value			η_p_^2^			*F* (*df*)				*P* value			η_p_^2^	
**Sex**										4.80 (1.98, 7705.74)			.008			0.001			13.73 (1.00, 3890.00)			<.001			0.004			22.71 (1.98, 7705.74)				<.001			0.006
	Male	23.98 (5.55)		24.19 (5.72)		24.22 (5.68)																													
	Female	23.13 (5.96)		23.75 (6.14)		23.57 (6.23)																													
**Age (y)**										1.17 (5.94, 7702.10)			.32			0.001			69.40 (3.00, 3888.00)			<.001			0.051			12.57 (1.98, 7702.10)				<.001			0.003
	18-29	23.91 (5.73)		24.35 (5.60)		24.37 (5.78)																													
	30-49	24.37 (5.76)		24.96 (5.91)		24.80 (5.82)																													
	50-64	23.79 (5.61)		24.09 (5.80)		24.03 (5.96)																													
	≥65	21.07 (5.38)		21.24 (5.49)		21.34 (5.58)																													
**Occupation**										0.49 (7.92, 7700.42)			.87			0.000			12.54 (4.00, 3887.00)			<.001			0.013			6.24 (1.98, 7700.42)				.002			0.002
	Employed	23.76 (5.51)		24.13 (5.70)		24.12 (5.69)																													
	Homemaker	22.26 (6.24)		22.78 (6.42)		22.52 (6.41)																													
	Student	23.70 (4.68)		24.24 (4.70)		24.33 (5.64)																													
	Unemployed	23.81 (6.33)		24.15 (6.28)		23.99 (6.41)																													
	Other	25.02 (5.97)		25.79 (6.41)		25.58 (6.53)																													
**Marital status**										0.55 (1.98, 7706.43)			.58			0.000			286.11 (1.00, 3890.00)			<.001			0.069			20.26 (1.98, 7706.43)				<.001			0.005
	Married	22.53 (5.41)		22.90 (5.55)		22.88 (5.57)																													
	Unmarried	25.46 (5.88)		25.91 (6.07)		25.75 (6.16)																													
**The presence of child**										0.15 (1.98, 7706.66)			.86			0.000			244.19 (1.00, 3890.00)			<.001			0.059			21.54 (1.98, 7706.66)				<.001			0.006
	Yes	22.44 (5.41)		22.82 (5.65)		22.74 (5.62)																													
	No	25.03 (5.86)		25.46 (5.93)		25.41 (6.03)																													
**Annual household income (in million; JPY^a^)**										0.72 (7.93, 5523.82)			.68			0.001			29.91 (4.00, 2786.00)			<.001			0.041			7.76 (1.98, 5523.82)				<.001			0.003
	<2.0	26.85 (6.38)		27.03 (6.29)		26.96 (6.60)																													
	2.0-3.9	24.12 (5.92)		24.62 (6.11)		24.42 (6.04)																													
	4.0-5.9	23.45 (5.67)		23.82 (5.77)		23.62 (5.74)																													
	6.0-7.9	23.21 (5.49)		23.54 (5.88)		23.75 (5.88)																													
	≥8.0	22.45 (5.58)		22.68 (5.65)		22.66 (5.81)																													

^a^1 JPY=US $0.0093.

### Psychosocial and Physical Factors Associated With Loneliness in the 3 Phases

Multimedia Appendix 2 shows the results of the linear mixed model analysis of the psychosocial and physical factors associated with loneliness in the 3 phases. Multiple regression analyses were performed for each phase, and explanatory variables significantly associated with UCLA-LS3 scores were applied in a linear mixed model. Sex (*F*_1.00,3398.03_=56.95; *P*<.001), marital status (*F*_1.00,4615.04_=36.45; *P*<.001), age (*F*_1.00,3568.70_=57.1; *P*<.001), K6 (*F*_1.00,8293.87_=76.37; *P*<.001), PHQ-9 (*F*_1.00,8474.12_=109.29; *P*<.001), SSS-8 (*F*_1.00,9340.97_=9.17; *P*=.002), LSNS-6 (*F*_1.00,9187.38_=1173.01; *P*<.001), exercise (*F*_1.00,9290.45_=14.06; *P*<.001), offline (*F*_1.00,8284.40_=82.46; *P*<.001), and web-based (*F*_1.00,8645.18_=60.46; *P*<.001) interaction with familiar people, optimism (*F*_1.00,8757.93_=147.41; *P*<.001), deterioration of household economy (*F*_1.00, 9023.79_=28.80; *P*<.001), deterioration of relationship with familiar people (*F*_1.00,8259.46_=80.30; *P*<.001), and frustration (*F*_1.00,8622.65_=54.36; *P*<.001) had significant effects on the UCLA-LS3 scores. Marital status (*F*_2.00,5964.59_=3.17; *P*=.04), LSNS-6 (*F*_2.00,6373.94_=3.53; *P*=.03), altruistic preventive behavior (*F*_2.00,6781.67_=10.87; *P*<.001), optimism (*F*_2.00,6813.54_=3.06; *P*=.047), and deterioration of household economy (*F*_2.00,6673.78_=3.76, *P*=.02) showed significant interactions with phases. Regarding marital status, the unmarried group had significantly higher scores on the UCLA-LS3 in all phases and showed a significant increase in the scores between phases 1 and 2. The married group showed a significant increase in the score between all phases. The altruistic preventive behavior scores in phases 1 and 2 were more negatively related to the UCLA-LS3 score than in phase 3. The optimism scores in phase 3 were more negatively related to the UCLA-LS3 scores than phases 1 and 2 scores. The LSNS-6 scores and deterioration of the household economy did not show a significant difference in the relationship with the UCLA-LS3 between the phases.

## Discussion

### Change of Loneliness and Social Isolation for 2 Years

While many psychosocial and physical variables showed improvement for 2 years, loneliness, social isolation, and the relationship with familiar people deteriorated, and the opportunities for exercise, favorite activities, and web-based interaction with familiar people decreased. Both loneliness and social isolation were severe compared with the results of studies conducted before the COVID-19 pandemic. In previous studies conducted during the prepandemic period in Japan, the mean score of UCLA-LS3 was 17.5 points and that of the LSNS-6 was 16.2 points [15,17]. The deterioration or lack of improvement over the past 2 years is a serious problem. Our results were consistent with previous studies in other countries to some extent; a systematic review showed that loneliness did not significantly improve during the pandemic [35], and a German study [36] reported that depression increased from the prepandemic to the first wave (June 2020), but declined in the second pandemic wave; loneliness increased during the first and second waves of the pandemic (January and February 2021). While people adapted to the repeated declarations of the state of emergency and the wave of increasing COVID-19 infections, there may have been an increased number of cases, exacerbating relationship problems. Future research comparing this region with areas that were not in a declared state of emergency could clarify this impact.

### The Relationship Between Social Isolation and Psychosocial and Physical Variables

Approximately half of the individuals experiencing social isolation in phase 1 remained socially isolated throughout the 2-year period. In addition, the presence or absence of social isolation over the 2-year period shifted to some extent within individuals, and more people developed social isolation than those who were able to resolve it. Thus, many people were unable to escape long-term social isolation during the pandemic, and in many cases, the scarcity of social networks became more severe over a long period. The results indicate that the problem of social isolation during the pandemic must be identified and addressed from a long-term perspective.

There were no notable differences in the association between social isolation and demographic characteristics between the phases, and significantly more men, people in the 50 to 64 age group, unemployed, unmarried, childless, and those with a household income below ¥JPY 3.9 million were socially isolated in all phases. Regarding the association between psychosocial and physical variables, in addition to worsening general physical and mental indicators such as loneliness, psychological distress, depression, and physical symptoms, they also had problems with lifestyle (exercise, diet, and sleep), frustration, deteriorated interpersonal relationships, decreased social interaction (both web-based and offline), decreased favorite activities, and a negative outlook for the future. They also exhibited fewer preventive behaviors to avoid infecting others, which may be an effect of fewer opportunities for interaction. The effect size was very small, except in phase 3, although it was significant for higher household financial deterioration. Anxiety and sleep problems related to COVID-19, lack of daily necessities, and difficulties in work and study, which were not significantly different between the groups in phases 1 and 2, were significantly different between the groups in phase 3; however, the effect size was very small. These results indicate that during the 2 years of the pandemic (whether under a declared state of emergency or not), the factors associated with a severe and persistent lack of social networks did not change significantly, indicating the need to strengthen isolation measures, especially for specific genders, age groups, income groups, and family structures, and to assess and improve physical and mental status, lifestyle habits, and social network interventions.

### The Relationship Between Loneliness and Psychosocial and Physical Variables

In all phases, loneliness was significantly higher among males, the 50 to 64 age group, the childless group, and the group with annual household incomes <JPY 2 million; this is similar to the results of social isolation. In terms of occupation, loneliness was high in the “other” group, which did not fit into any occupation category, but this result was difficult to interpret because the kind of employment status was unclear.

**Figure 3:**
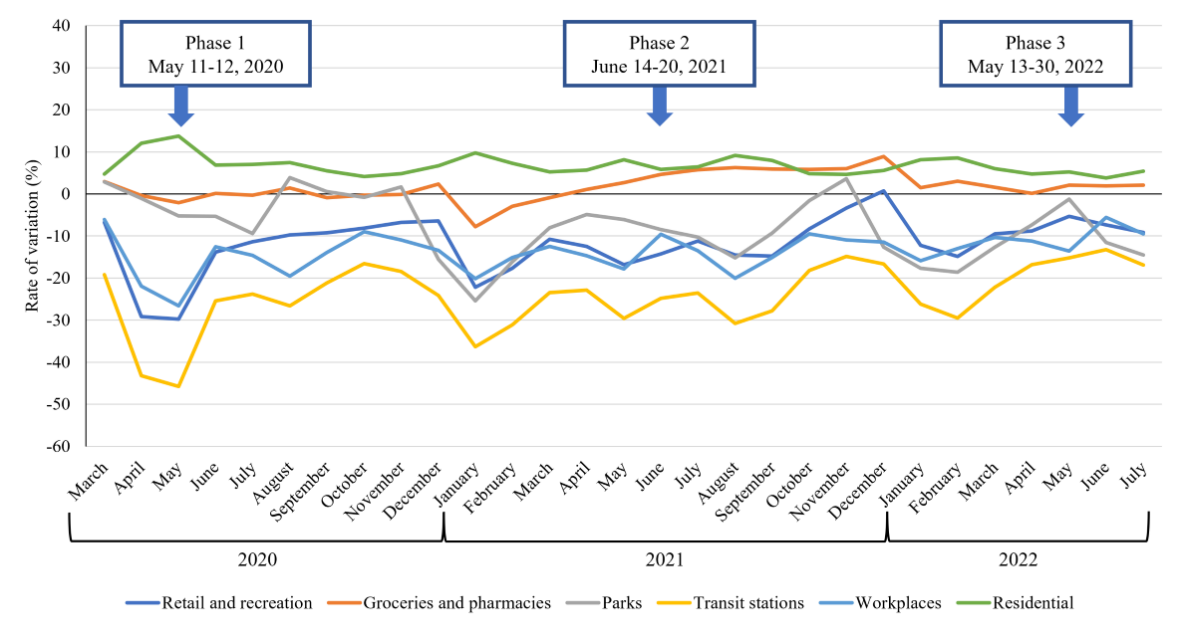
Movement trends in Japan during COVID-19 pandemic The data from “COVID-19 Community Mobility Report” by Google shows how visitors to (or time spent in) categorized places changed compared with the baseline days. A baseline day represents a normal value on the day of the week. The baseline day was the median value from the 5 week period of January 3, 2020, to February 6, 2020.

The results of the linear mixed model analysis showed that most psychosocial and physical variables were related to loneliness regardless of the phase. Regarding the variables that showed a significant interaction with the phases, increased altruistic preventive behavior and a negative outlook for the future were more strongly associated with severe loneliness in phase 3. Among the variables that had significant interactions with the phases, the LSNS-6 score showed no significant differences in association by phase; however, the association between fewer social networks and stronger loneliness tended to be more pronounced in phase 2. In addition, although the interaction was not significant, the association between fewer face-to-face interactions, worse relationships with familiar people, and worse loneliness tended to be stronger in phase 3. The factors that increase loneliness during a pandemic may become more varied and complex over time. Figure 3 indicates mobility changes in Japan during the COVID-19 pandemic by geography across different categories of places such as retail and recreation, groceries and pharmacies, parks, transit stations, workplaces, and residential areas (Google LLC) [37]. This figure shows that phase 1 was a period of significant decrease in travel to retail or recreation, transit stations, and workplaces, and this phenomenon normalized from phases 2 and 3. Under a declared state of emergency for COVID-19 (ie, the first half of the survey period), the environmental changes caused by following social demands led to social isolation, and loneliness was intensified. However, even if the life changes associated with the social situation later eased, interpersonal interactions and relationships did not improve, and the lack of positive thinking due to the prolonged pandemic may have exacerbated loneliness and social isolation. Regarding altruistic preventive behavior, another longitudinal survey [38] reported that an increase in physical isolation was only present for people with high COVID-19 concerns during the pandemic, in contrast to the early part of the pandemic in Japan, when many people refrained from going out. The latter part of the survey period was a time when people’s behavior was changing toward normalization, and those who were cautious about infection may have increased their sense of isolation by missing parties and other activities with their peers.

As this study and other previous studies have shown, the problems of loneliness and social isolation have remained unresolved during the long-term COVID-19 pandemic; therefore, they should be urgently addressed to protect people’s mental health. A systematic review of interventions to reduce social isolation and loneliness during the COVID-19 physical distancing measures [39] indicated that the most effective interventions for loneliness involved either cognitive or educational components or facilitated communication and networking between peers. Although there were few effective interventions for social isolation, it was stated that remote intervention could be effective. By establishing a system that provides web-based interventions that can effectively and directly relieve loneliness while simultaneously improving the factors associated with loneliness that have changed over time since the start of the pandemic and with varying social conditions, as identified in this study, we can prepare for the social isolation and loneliness that could occur in future pandemics. In particular, this study showed that a variety of factors were more strongly related to loneliness and were more complex in the last survey phase than in the earlier phases, suggesting that preventive interventions may be important in the early stages of a pandemic to efficiently improve loneliness and social isolation. Web-based interventions may be particularly useful in the early stages of a pandemic when the virus is not well-characterized and people are strongly urged to refrain from going out.

### Limitations

This study has several limitations. First, as the data were collected through a web-based survey, random sampling was not conducted. Therefore, we cannot guarantee the representativeness of the sample, as it cannot be matched to the percentages of each age group or sex in each region. In addition, people registered with web-based survey companies may be more willing to participate in surveys than those who are not. They may have social networks through which they can obtain information on such cooperation. The population not registered with the survey firm may have more severely socially isolated individuals who may have different characteristics and require additional support. Regarding the family environment, for example, the 2020 Census reported that 58.6% of the Japanese population aged ≥20 years were married [40]. While 63.95% (2489/3892) of the participants in this study were married, which is comparatively high, the small number of older participants who were bereaved of spouses suggests that this was not a particularly large proportion of married people. From this perspective, the living environments of the registrants of the survey company, who formed the study population, may not have been significantly different, in relation to social isolation or loneliness, from those of the nonregistrant population. However, most previous studies conducted in Japan during the COVID-19 pandemic using the LSNS-6 and the UCLA were conducted on survey company registrants, and it is difficult to rigorously compare these results with a nonregistered population. It is necessary to interpret these results by considering the possibility that experiences of isolation and loneliness may differ between registrants and nonregistrants. Second, significant differences between individuals who participated in the 3 phases and those who did not participate in phases 2 or 3 were indicated for some sociodemographic characteristics and psychological variables.

### Conclusions

The results of this study indicate that the problems of loneliness and social isolation remain unresolved during the long-term COVID-19 pandemic. While nearly half of the social isolation in the early phase of the pandemic persisted throughout the 2-year period, more people developed social isolation than those who were able to resolve it. Demographic characteristics (male sex, the 50-64 age group, lower income, etc.) and psychosocial variables (psychological distress, lifestyle, relationship, and interaction with familiar people), which were related to social isolation, were consistent for the 2 years. In addition, factors that increased loneliness during the pandemic became more varied and complex over time. By establishing a system that provides interventions that can effectively relieve loneliness by considering the factors associated with loneliness that have changed over time since the start of the pandemic and with varying social conditions, we may be able to better prepare for social isolation and loneliness that could occur in future pandemics.

## Appendix

Multimedia Appendix 1 Items about lifestyle, coping behavior, and stressors related to the COVID-19 pandemic.

Multimedia Appendix 2 Results of linear mixed model analysis of psychosocial and physical factors associated with loneliness in the 3 phases.

## Abbreviations

K6: Kessler Psychological Distress Scale-6
LSNS-6: Lubben Social Network Scale-6
PHQ-9: Patient Health Questionnaire-9
SSS-8: Somatic Symptom Scale-8
UCLA-LS3: University of California, Los Angeles Loneliness Scale Version 3
