# Supplementary material for: Loneliness and Social Isolation Factors Under the Prolonged COVID-19 Pandemic in Japan: 2-Year Longitudinal Study
Source: JMIR Public Health Surveill. 2024 Sep 9;10:e51653. doi: 10.2196/51653 (PMC11420607; doi:10.2196/51653)
Supplement: Multimedia Appendix 1 [file publichealth_v10i1e51653_app1.docx]

Appendix 1. Items about lifestyle, coping behavior, and stressors related to COVID-19 pandemic

| 1. | I exercised for my health (whether indoors or outdoors). |
| --- | --- |
| 2. | I took meals considering the nutrition balance. |
| 3. | I kept regular awakening time and bedtime approximately. |
| 4. | I engaged in activities such as hobbies with absorbing interest. |
| 5. | I interacted with my family or friends on a face-to-face basis (outside of work or class). |
| 6. | I interacted with my family or friends online using chat or video calling (except work or class). |
| 7. | I spontaneously refrained from going out or took preventive behaviors (e.g., wearing a mask) to prevent coronavirus disease 2019 infection to my family or other people. |
| 8. | I thought about the future positively. |
| 9. | The family budget has tightened. |
| 10. | A personal relationship with a close person such as family or friends got worse. |
| 11. | I have become easily annoyed or irate due to life changes. |
| 12. | I felt nervous or anxious when I watched the news about coronavirus disease 2019. |
| 13. | Because I kept thinking about coronavirus disease 2019 infection, I could not sleep. |
| 14. | My daily life was disrupted due to the shortage of materials relating to prevention for coronavirus disease 2019 infection (e.g., mask or thermometer) or other daily supplies. |
| 15. | My work or schoolwork was disrupted due to a life change. |
