# Supplementary material for: Loneliness and Social Isolation Factors Under the Prolonged COVID-19 Pandemic in Japan: 2-Year Longitudinal Study
Source: JMIR Public Health Surveill. 2024 Sep 9;10:e51653. doi: 10.2196/51653 (PMC11420607; doi:10.2196/51653)
Supplement: Multimedia Appendix 2 [file publichealth_v10i1e51653_app2.docx]

Appendix 2. Results of linear mixed model analysis of psychosocial and physical factors associated with loneliness in the 3 phases.

|  | | Estimate | SE | *t* (df) | *P* value | 95% CI |
| --- | --- | --- | --- | --- | --- | --- |
| Intercept | | 27.69 | 0.44 | 63.34 (9133.34) | <.001 | 26.84-28.55 |
| Phase (ref: phase 3) | | | | | | |
|  | Phase 1 | −0.46 | 0.53 | −0.88 (6449.76) | .38 | −1.50 to 0.57 |
|  | Phase 2 | −0.22 | 0.51 | −0.44 (6362.03) | .66 | −1.21 to 0.77 |
| Sex (ref: female) | | | | | | |
|  | Male | 0.95 | 0.16 | 6.12 (6943.72) | <.001 | 0.65-1.25 |
| Marital status (ref: married) | | | | | | |
|  | Unmarried | 0.53 | 0.17 | 3.20 (8443.45) | .001 | 0.21-0.86 |
| Annual household income (in million; JPY; ref: ≥8.0 million) | | | | | | |
|  | <2.0 | 0.47 | 0.27 | 1.76 (9371.74) | .08 | −0.05 to 1.00 |
|  | 2.0-3.9 | −0.01 | 0.20 | −0.06 (9371.78) | .95 | −0.40 to 0.38 |
|  | 4.0-5.9 | −0.20 | 0.19 | −1.10 (9358.86) | .27 | −0.57 to 0.16 |
|  | 6.0-7.9 | −0.08 | 0.20 | −0.40 (8826.61) | .69 | −0.46 to 0.31 |
| Age (years) | | −0.04 | 0.01 | −6.25 (7374.22) | <.001 | −0.05 to −0.03 |
| K6^a^ | | 0.08 | 0.02 | 4.60 (7929.74) | <.001 | 0.05-0.12 |
| PHQ-9^b^ | | 0.10 | 0.02 | 5.02 (7855.28) | <.001 | 0.06-0.14 |
| SSS-8^c^ | | 0.05 | 0.02 | 3.04 (8490.79) | .002 | 0.02-0.08 |
| LSNS-6^d^ | | −0.28 | 0.01 | −23.18 (8835.37) | <.001 | −0.30 to −0.26 |
| Exercise | | −0.12 | 0.04 | −3.21 (8308.67) | .001 | −0.19 to −0.05 |
| Offline interaction with familiar people | | −0.29 | 0.04 | −7.08 (7755.03) | <.001 | −0.37 to −0.21 |
| Web-based interaction with familiar people | | −0.16 | 0.04 | −4.36 (7897.14) | <.001 | −0.24 to −0.09 |
| Altruistic preventive behavior | | 0.15 | 0.04 | 3.64 (8062.71) | <.001 | 0.07-0.23 |
| Optimism | | −0.47 | 0.05 | −9.33 (7952.15) | <.001 | −0.57 to −0.37 |
| Deterioration of household economy | | 0.14 | 0.04 | 3.19 (7988.27) | .001 | 0.05-0.22 |
| Deterioration of relationship with familiar people | | 0.38 | 0.06 | 6.90 (7736.60) | <.001 | 0.27-0.49 |
| Frustration | | 0.28 | 0.05 | 5.40 (7940.34) | <.001 | 0.18-0.38 |
| Difficulty owing to the lack of daily necessities | | 0.03 | 0.05 | 0.61 (7699.49) | .54 | −0.07 to 0.13 |
| Phase × sex (ref: phase 3 and female) | | | | | | |
|  | Phase 1 × male | 0.07 | 0.16 | 0.42 (6033.67) | .67 | −0.25 to 0.39 |
|  | Phase 2 × male | −0.08 | 0.16 | −0.47 (5960.76) | .64 | −0.39 to 0.24 |
| Phase × marital status (ref: phase 3 and married) | | | | | | |
|  | Phase 1 × unmarried | 0.34 | 0.18 | 1.87 (6164.11) | .06 | −0.02 to 0.70 |
|  | Phase 2 × unmarried | 0.43 | 0.18 | 2.41 (6057.67) | .02 | 0.08-0.78 |
| Phase × annual household income (JPY) (ref: phase 3 and ≥8.0 million) | | | | | | |
|  | Phase 1 × <2.0 million | 0.13 | 0.34 | 0.39 (6503.52) | .69 | −0.53 to 0.79 |
|  | Phase 1 × 2.0-3.9 million | 0.38 | 0.23 | 1.61 (6354.12) | .11 | −0.08 to 0.84 |
|  | Phase 1 × 4.0-5.9 million | 0.46 | 0.22 | 2.08 (6353.38) | .04 | 0.03-0.89 |
|  | Phase 1 × 6.0-7.9 million | 0.15 | 0.25 | 0.60 (6565.38) | .55 | −0.33 to 0.63 |
|  | Phase 2 × <2.0 million | −0.18 | 0.32 | −0.57 (6353.39) | .57 | −0.82 to 0.45 |
|  | Phase 2 × 2.0-3.9 million | 0.27 | 0.23 | 1.19 (6177.12) | .23 | −0.18 to 0.72 |
|  | Phase 2 × 4.0-5.9 million | 0.17 | 0.22 | 0.80 (6199.08) | .42 | −0.25 to 0.59 |
|  | Phase 2 × 6.0-7.9 million | 0.05 | 0.24 | 0.21 (6402.64) | .83 | −0.43 to 0.53 |
| Phase × age (ref: phase 3) | | | | | | |
|  | Phase 1 | 0.00 | 0.01 | 0.15 (6158.25) | .88 | −0.01 to 0.01 |
|  | Phase 2 | 0.00 | 0.01 | 0.50 (6062.95) | .61 | −0.01 to 0.02 |
| Phase × K6 (ref: phase 3) | | | | | | |
|  | Phase 1 | 0.05 | 0.03 | 2.08 (7224.69) | .04 | 0.00-0.11 |
|  | Phase 2 | 0.00 | 0.03 | 0.10 (7121.87) | .92 | −0.05 to 0.05 |
| Phase × PHQ-9 (ref: phase 3) | | | | | | |
|  | Phase 1 | 0.04 | 0.03 | 1.57 (7131.18) | .12 | −0.01 to 0.10 |
|  | Phase 2 | 0.05 | 0.03 | 1.80 (7070.37) | .07 | 0.00-0.11 |
| Phase × SSS-8 (ref: phase 3) | | | | | | |
|  | Phase 1 | −0.02 | 0.02 | −0.97 (6810.82) | .33 | −0.06 to 0.02 |
|  | Phase 2 | −0.03 | 0.02 | −1.53 (6627.85) | .13 | −0.07 to 0.01 |
| Phase × LSNS-6 (ref: phase 3) | | | | | | |
|  | Phase 1 | 0.01 | 0.02 | 0.63 (6494.36) | .53 | −0.02 to 0.04 |
|  | Phase 2 | −0.03 | 0.01 | −1.88 (6417.76) | .06 | −0.06 to 0.00 |
| Phase × exercise (ref: phase 3) | | | | | | |
|  | Phase 1 | 0.04 | 0.05 | 0.86 (6717.85) | .39 | −0.05 to 0.14 |
|  | Phase 2 | 0.03 | 0.05 | 0.55 (6478.44) | .59 | −0.07 to 0.12 |
| Phase × offline interaction with familiar people (ref: phase 3) | | | | | | |
|  | Phase 1 | 0.12 | 0.05 | 2.24 (7150.15) | .03 | 0.01-0.23 |
|  | Phase 2 | 0.10 | 0.05 | 1.90 (6994.10) | .06 | 0.00-0.21 |
| Phase × Web-based interaction with familiar people (ref: phase 3) | | | | | | |
|  | Phase 1 | −0.03 | 0.05 | −0.61 (7028.73) | .54 | −0.13 to 0.07 |
|  | Phase 2 | −0.04 | 0.05 | −0.71 (6813.47) | .48 | −0.14 to 0.06 |
| Phase × altruistic preventive behavior (ref: phase 3) | | | | | | |
|  | Phase 1 | −0.25 | 0.05 | −4.65 (6931.51) | <.001 | −0.36 to −0.15 |
|  | Phase 2 | −0.14 | 0.05 | −2.59 (6766.53) | .01 | −0.25 to −0.03 |
| Phase × optimism (ref: phase 3) | | | | | | |
|  | Phase 1 | 0.17 | 0.07 | 2.47 (7012.74) | .01 | 0.03-0.30 |
|  | Phase 2 | 0.09 | 0.07 | 1.34 (6842.49) | .18 | −0.04 to 0.22 |
| Phase × deterioration of household economy (ref: phase 3) | | | | | | |
|  | Phase 1 | −0.06 | 0.06 | −1.16 (6859.04) | .25 | −0.17 to 0.04 |
|  | Phase 2 | 0.08 | 0.06 | 1.45 (6650.53) | .15 | −0.03 to 0.19 |
| Phase × deterioration of relationship with familiar people (ref: phase 3) | | | | | | |
|  | Phase 1 | −0.13 | 0.07 | −1.83 (7123.93) | .07 | −0.28 to 0.01 |
|  | Phase 2 | −0.14 | 0.07 | −1.97 (6981.03) | .049 | −0.29 to 0.00 |
| Phase × frustration (ref: phase 3) | | | | | | |
|  | Phase 1 | −0.11 | 0.07 | −1.69 (7087.73) | .09 | −0.25 to 0.02 |
|  | Phase 2 | −0.03 | 0.07 | −0.42 (6872.53) | .68 | −0.16 to 0.10 |
| Phase × difficulty owing to the lack of daily necessities (ref: phase 3) | | | | | | |
|  | Phase 1 | 0.04 | 0.06 | 0.59 (7276.40) | .55 | −0.09 to 0.16 |
|  | Phase 2 | 0.02 | 0.07 | 0.35 (6887.68) | .73 | −0.11 to 0.16 |

^a^K6: Kessler Psychological Distress Scale-6.

^b^PHQ-9: Patient Health Questionnaire-9.

^c^SSS-8: Somatic Symptom Scale-8.

^d^LSNS-6: Lubben Social Network Scale.
